# Ectrodactyly-Ectodermal Dysplasia Clefting Syndrome: A Case Report of Its Dental Management with 2 Years Follow-Up

**DOI:** 10.1155/2020/8418725

**Published:** 2020-03-23

**Authors:** Anamika Bharati, Saumya Navit, Suleman Abbas Khan, Seema Jabeen, Nishi Grover, Meenakshi Upadhyay

**Affiliations:** Department of Pediatric and Preventive Dentistry, Saraswati Dental College, Lucknow, U.P, India

## Abstract

Ectrodactyly-ectodermal dysplasia clefting syndrome is a rare genetic disorder characterized by the triad of ectrodactyly-ectodermal dysplasia and facial clefting of lip or palate or both along with some systemic manifestations. Although each defect that comprises the syndrome has been known to occur as a separate entity, the congregation of all three anomalies in a single individual appears to be an extremely rare occurrence, with incidence being approximately 1.5/100 million population. Early diagnosis and management of clinical manifestations associated with ectrodactyly-ectodermal dysplasia clefting syndrome present a unique challenge. We report a case of this rare disorder in an 11-year-old male patient along with its dental management using a multidisciplinary approach.

## 1. Introduction

Ectrodactyly-ectodermal dysplasia clefting (EEC) syndrome is an autosomal dominant disorder associated mainly with a triad of cardinal signs ectrodactyly, ectodermal dysplasia, and cleft lip/palate [1]. It was first documented by Eckoldt and Martens in 1804 [2] and the term EEC syndrome was coined by Rudiger et al. in 1970 [1]. Ectrodactyly or split hand/split foot malformation (SHFM), by strict definition, is the congenital absence of central rays of limbs [3]. It was initially documented in 1770 among a tribe of Guiana Indians [3]. It is often found with syndactyly [4]. Ectodermal dysplasia consists of a large heterogeneous group of inherited disorders. It comprises primary defects in the development of 2 or more tissues derived from the embryonic ectoderm and can present as hypoplastic teeth, dystrophic nails, dry skin, or sparse hair with occasional lacrimal duct obstruction [5]. Clefting may affect the lip and/or palate. Ectrodactyly, ectodermal dysplasia, and clefting, all three rarely coexist in a single individual with incidence being 1.5/100 million population [6]. Hearing loss, lacrimal duct defect, genitourinary defect, delayed developmental milestones, malignant lymphoma, and occasional mental retardation are some other features associated with EEC syndrome [7]. A review of 230 cases from English, German, French, Italian, and Dutch publications found EEC syndrome to include ectrodactyly (84%), ectodermal dysplasia (77%), cleft lip and/or palate (68%), lacrimal tract abnormalities (59%), urogenital abnormalities (23%), and conductive hearing loss (14%). The review of 230 cases identified 116 as familial and 114 as sporadic [8]. We report a sporadic case of this disorder in an 11-year-old male who had the classical split-hand/split-foot malformation of all 4 limbs and ectodermal abnormalities with operated cleft lip and palate on the right side of the face.

## 2. Case Report

**Figure 1:**
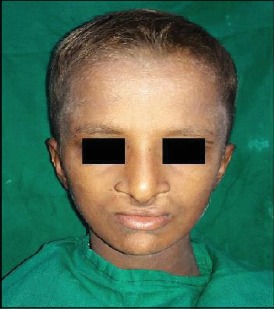
Patient with fine, lightly pigmented, sparse hair and dry and rough skin with scanty eyebrows.

**Figure 2:**
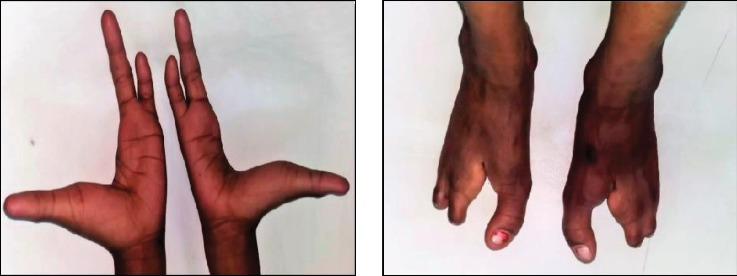
Upper limbs and lower limbs showing ectrodactyly with absent 2^nd^ and 3^rd^ digit/toes.

**Figure 3:**
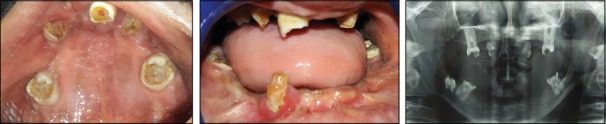
Intraoral and radiographic examination revealing oligodontia with 11, 12, 16, 21, 22, 26, 36, 42, and 46 as the teeth present. Unerupted tooth 47 present in the lower jaw.

**Figure 4:**
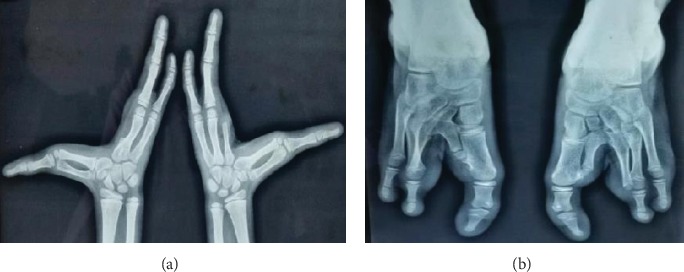
(a) Radiograph of a hand showing syndadactyly of 1^st^ and 2^nd^ metacarpals with aplasia of 2^nd^ and hypoplasia of 3^rd^ phalange. (b) Radiograph of a foot showing syndactyly of 1^st^ and 2^nd^ metatarsals with aplasia of 2^nd^ and hypoplasia of 3^rd^ phalanges.

An 11-year-old male patient reported to the Department of Pedodontics with the chief complaint of decayed and missing teeth in the upper and lower arches. He also complained of limb anomalies. Past medical history revealed that the patient had a cleft lip and palate on the right side of the face at the time of birth which was operated at the age of 6 months and 3 years, respectively. He also gave history of sticky discharge from eyes and decreased sweating and recurrent fever during summer. The patient was the youngest of the three siblings; the older two were unaffected. No similar family history was noted. The patient was born of a nonconsanguineous marriage. On physical examination, he was found to have fine, lightly pigmented, sparse hair and dry and rough skin with scanty eyebrows (Figure 1). Both upper limbs and lower limbs had ectrodactyly with absent 2^nd^ and 3^rd^ digit/toes (Figure 2). The intraoral examination revealed oligodontia with 11, 12, 16, 21, 22, 26, 36, 42, and 46 as the only teeth present (Figure 3). There was grade I mobility present with respect to 11, 12, 21, and 22; grade III mobility was present with respect to 42. The patient presented with defective tooth enamel, thin alveolar ridge, and reduced vertical bone height in both the upper and lower jaws. Panoramic view revealed an unerupted tooth 47 in the lower jaw (Figure 3). Radiographic evaluation of the hand and foot showed median cleft with variable degrees of aplasia/hypoplasia suggestive of ectrodactyly. Syndadactyly of the 1^st^ and 2^nd^ metacarpals with aplasia of the 2^nd^ and hypoplasia of the 3^rd^ phalange was seen in the hand (Figure 4(a)). Similarly, syndactyly of the 1^st^ and 2^nd^ metatarsals with aplasia of the 2^nd^ and hypoplasia of the 3^rd^ phalanges were seen in the foot radiograph (Figure 4(b)). Based on the history, clinical features, and radiographic examination, the child was diagnosed as a case of ectrodactyly-ectodermal dysplasia-cleft (EEC) syndrome. In this case, due to unfavourable tongue positions, muscle attachments, and high palatal vault, which render the stability and retention of the prosthesis difficult, overdenture was one of the most practical measures. Treatment option preferred was noncoping complete maxillary and mandibular overdenture. Informed consent was obtained for the agreed treatment. The treatment planned was completed in four phases.

### 2.1. Phase I. Oral Prophylaxis and Fluoride Application.

The patient had an oral prophylaxis carried out with hand scalers to remove calculus and stain from his teeth. The patient's teeth were rubber cup polished with fine paste, and fluoride varnish was applied on his teeth. He was instructed not to eat or drink anything for 30 minutes. After 30 minutes, the patient was instructed to have only soft food and not to brush his teeth until the next morning—to give the varnish optimal time to work.

At the recall appointment, the patient was given extensive homecare instructions that included reviewing his brushing and flossing. Due to deformity of his hands, he was using a toothbrush with a small ball attached at the bottom to help him brush. The patient was asked to demonstrate his brushing technique and modifications were made to help him cover more of his tooth surfaces and remove more plaque. Other recommendations made were daily use of a fluoride toothpaste with concentration of 5000 ppm.

### 2.2. Phase II. Extraction of Grade III mobile tooth 42 was done.

### 2.3. Phase III

**Figure 5:**
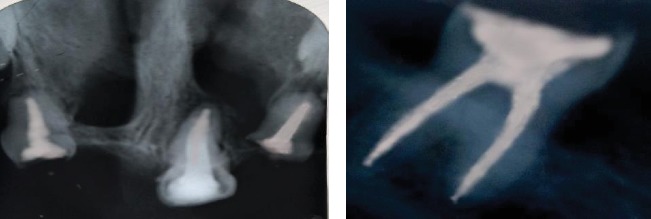
Postoperative radiograph showing root canal treated teeth with respect to teeth 11,21,22 and 46.

Root canal treatment with respect to teeth 11, 12, 16, 21, 22, 26, 36, and 46 was done, followed by restoration with composite resin (Figure 5). Coronal modification of the teeth was carried out to act as abutments for the overdenture.

### 2.4. Phase IV: Prosthetic Rehabilitation

**Figure 6:**
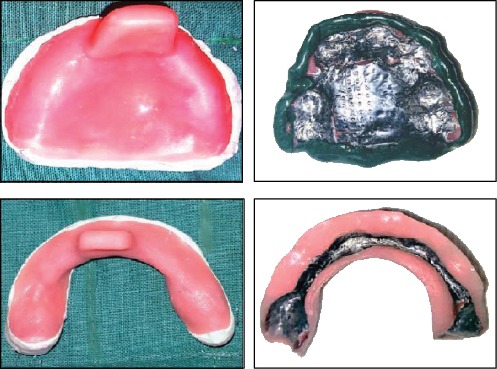
Special trays fabricated on the cast model with self-cure acrylic resin. Aluminium foil has been used for making the spacer.

**Figure 7:**
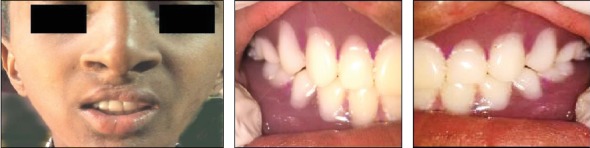
The complete overdentures delivered to the patient. Frontal and lateral views of the denture inside the patient's mouth.

Primary impression for the maxillary and mandibular arches was made. Special trays were fabricated on the cast model with self-cure acrylic resin (Figure 6). As the tissue was fragile, the spacer was made using aluminium foil. Maxillary and mandibular final impressions were made with regular body elastomer. Master casts were prepared. Tentative jaw relation was taken which was followed by tooth setting and try-in. The denture was processed into heat cure denture base acrylic. The complete overdentures were delivered (Figure 7). Patient was motivated for denture wearing and postinsertion instruction on the maintenance of oral hygiene and dentures was given. Fluoride application was done on the abutments. Recall appointments were scheduled after 1 and 4 weeks. Patient was advised for using artificial saliva (ICPA Wet Mouth) thrice a day for maintaining the moist environment inside the oral cavity. At recall appointments, no pressure spots were noticed, and static and dynamic occlusions showed no interferences. Retention was excellent, and the parents reported significant improvement in his speech and mastication. The increased self-esteem improved the socialization skills of the boy. Follow-up visits were scheduled after every 3 months so as to accommodate growth and development.

### 2.5. Assessment of Oral Health-Related Quality of Life

Oral health-related quality of life (OHRQoL) was assessed at the beginning of the treatment and at 6 monthly recall visits using a structured (16-item) child perception questionnaire (CPQ11-14). The questionnaire consisted of four domains: oral symptoms (*n* = 4), functional limitations (*n* = 4), emotional wellbeing (*n* = 4), and social wellbeing (*n* = 4). The questions assessed the frequency of events in the past 6 months regarding the child's oral/orofacial condition. The response options of each domain were recorded on a Likert scale from 0 to 4: never = 0, once/twice = 1, sometimes = 2, often = 3, and everyday/almost every day = 4. The overall score extended from 0 = perfect OHRQoL to 64 = worst OHRQoL. The scores after 1 year and 2 years posttreatment have been summarized in the Table 1.

**Figure 8:**
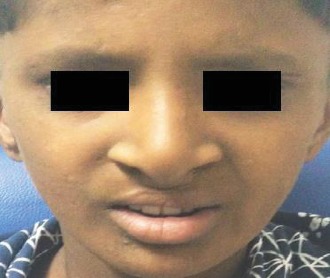
Photograph of the patient after 2 years follow-up.

After 2 years follow-up, it was observed that the child's quality of life was well improved in all the four assessed domains (Figure 8).

## 3. Discussion

There are two clinical forms in which EEC syndrome may exist: one with cleft lip with or without cleft palate and the other with cleft palate alone [9]. In this case, the patient was born with both cleft lip and cleft palate. This disorder has been attributed to mutations in a gene encoding p63 [10]. From the aspect of oral health, patients with EEC syndrome are more susceptible to caries and gingivitis. They often face difficultly in maintaining oral hygiene due to ectrodactyly and limitations of using the hands [11]. They may suffer from problems of oral/facial development, speech, swallowing, and esthetics due to missing, abnormally shaped, and malpositioned teeth. Since the dental manifestations of this syndrome persist throughout life, dentists should work meticulously for the age appropriate dental rehabilitation of the patient. The preservation of deciduous and permanent teeth should be done whenever possible to conserve the investing bone for support and retention of removable prostheses. Overdenture provides significant advantages biologically such as decreased bone resorption, increased masticatory performance, and directional sensitivity. Hence, in the present case report, we used tooth-supported overdenture as a viable option in contrast to other case reports [12–14].

Relining, rebasing, or remaking of the prosthesis is required to accommodate growth changes which we did at regular 6-month visits. A decrease in the secretory salivary levels increases the risk of caries after denture use. Therefore, fluoride application is very much important to protect the remaining teeth. The principal advantage of an overdenture is that the patient has the psychological benefit of having his own teeth which outweighs all the disadvantages stated. In a similar case report, Gupta et al. [15] used removable cast partial dentures for the management of the missing teeth in case of ectrodactyly, ectodermal dysplasia, and cleft lip/palate syndrome.

Comprehensive treatment aiming consultation with a child psychologist, speech therapist, dermatologist, plastic surgeon, ophthalmologist, and renal specialist should be provided to the patient as and when required. In this case, the patient was referred to ophthalmologist as the patient complained of dry eyes, sticky discharge from the eyes, and eyelids completely glued upon while waking in the morning. The patient was diagnosed with nasolacrimal duct obstruction. The patient was attended upon by a team of doctors which comprised a pedodontist, paediatrician, and plastic surgeon during his cleft lip and palate surgery at the age of 6 months and 3 years.

## 4. Conclusion

Early diagnosis and management of clinical manifestations associated with EEC syndrome requires a multipronged approach by a team consisting of physicians from several clinical modalities to provide comprehensive medical care. This disorder is of concern to dental practitioners, and we need to be aware of dental conditions presenting with the syndrome and its management. A sympathetic, rationale, and multidisciplinary approach is necessary to improve the physical, psychological, and social integration of such patients.

## Figures and Tables

**Table 1:** 

Domain	Before treatment	1 year post treatment	2 years post treatment
Oral symptoms	12/16	6/16	4/16
Functional limitation	11/16	7/16	4/16
Emotional wellbeing	13/16	4/16	1/16
Social wellbeing	11/16	5/16	2/16
Overall scale score	47/64	22/64	11/64
